# Dynamic mechanical properties of structural anisotropic coal under low and medium strain rates

**DOI:** 10.1371/journal.pone.0236802

**Published:** 2020-08-12

**Authors:** Minmin Li, Weimin Liang, Gaowei Yue, Xinjun Zheng, Heng Liu

**Affiliations:** School of Civil Engineering, Henan Polytechnic University, Jiaozuo, Henan, China; China University of Mining and Technology, CHINA

## Abstract

The static mechanical properties of coal rock show anisotropism, which makes the permeability have anisotropic characteristics partly. The dynamic impact mechanical characteristics of structural anisotropic coal under low and medium strain rates were studied by using self-made vertical Split Hopkinson Bar (SHPB) equipment. The peak stress, the strain rate, dynamic elastic modulus and failure characteristics of raw coal with three coring directions were analyzed under the influence of five impact loads and structural anisotropy. The peak stress increases linearly with impact load, and the maximum strain rate and the dynamic elastic modulus increase exponentially with impact load. The coal samples display anisotropic mechanical characteristics. The values of maximum strain rate, peak stress and dynamic elastic modulus are ranked with directions by the perpendicular to bedding direction (Z direction), the parallel to bedding direction (X direction), and the oblique 45° to bedding direction (Y direction). Dynamic mechanical properties of structural anisotropic coal provide a theoretical basis for gas seepage in far-blasting field.

## 1 Introduction

Gas drainage in coal mine could effectively prevent coal and gas outburst, but the low gas drainage efficiency of low-permeability coal takes a challenge for gas drainage [1]. For hard coal, the deep hole blasting is a conventional technology to generate cracks in coal seam, which is beneficial for the gas permeability and drainage efficiency [2]. The porous medium permeability is affected by the connection of pores and cracks [3–5]. The layout and optimization of blast holes have obtained significant attention and made acceptable achievements in the isotropy coal circumstances [6–8]. In fact, the coal seam has abundant bedding and cleat structures during long geological history, which partly leads to the anisotropy of coal body about the mechanical properties [9,10]. The internal structures of coal will be changed by dynamic load [11,12], and according to the failure characteristics of coal body under blasting, the damage scope can be roughly divided into four areas: enlarged cavity, crushing area, cracking area and vibration area. In the crushing area, most of the hard coal is crushed under the impact load. In the vibration area, the coal particle vibrates elastically, and the coal structure does not change after blasting. In the cracking area, radial and circumferential cracks appear in coal, forming macroscopic and microscopic cracks. The blasting will lead to invisible micro-cracks in the place far away from the deep hole blasting location, which is helpful to improve the permeability of coal body. Therefore, the dynamic mechanical failure properties of coal body under different impact loads are of great significance for determining the parameters of deep hole blasting.

The dynamic properties of coal body in the field test of deep hole blasting is difficult to be obtained, therefore, the indoor test methods of the dynamic mechanical properties were studied, such as Split Hopkinson Bar (SHPB), light gas gun test, pneumatic impact device, hammer penetrometer and plane oscilloscope generator. Among them, SHPB is a common device to test the strain rate in the range of 10^1^-10^3^ s^-1^, in which the strain rate represents general mechanical impact, blasting and explosion dynamic loads, etc. [13–15]. Some researchers improved the device to test the dynamic mechanical properties of coal rock for high strain rate (10^2^-10^4^ s^-1^) and medium strain rate (10^1^-10^2^ s^-1^) [16–21]. However, the dynamic mechanical properties of coal rock under low strain rate (0-10 s^-1^) are rarely studied. It is difficult for horizontal SHPB impact device to obtain stable low velocity and low strain rate at low impact pressure [22]. Therefore, the self-made vertical SHPB impact device, which combines the advantages of the drop hammer device and horizontal SHPB impact device, was adopted to obtain the dynamic mechanical characteristics of structural anisotropic coal.

Bedding and cleat in coal rock have great influence on its dynamic performance. Jeager [23] first proposed the concept of strength anisotropy of coal rock caused by single weak plane effect in 1960, and formed the single weak plane theory, which is the initial research achievement about the anisotropy of coal rock. At first, researchers tested the static mechanical properties of anisotropic coal rock, and pointed out that the compressive strength and tensile strength of coal rock have obvious anisotropic characteristics [24,25]. With the wide application of SHPB technology, researchers had tested the mechanical properties of anisotropic coal body under high strain rate by the SHPB impact device. J. R. Klepaczko et al. [26] analyzed the elastic and viscoelastic properties of coal at the different strain rates under impact load for the first time, determined that the elastic properties of coal show strain rate sensitivity under low and medium strain rates. Zhao et al. [27], Wang et al. [28] conducted an experimental study on dynamic fracture toughness of coal samples under impact load by using SHPB impact device, and discussed the effect of impact speed and bedding angle. Wu et al. [29] carried out the dynamic compression tests by using the SHPB impact device under different impact speeds for five different layered rocks with the bedding angles of 0°, 22.5°, 45°, 67.5° and 90°. The results showed that the dynamic compressive strength with the dip angle of 0^o^ is the highest, and with the dip angle of 67.5° is the lowest. Xie et al. [21] developed a comparative experiment on dynamic mechanical failure characteristics of raw coal samples in different coring direction and sandstone samples with good homogeneity, which showed significant differences in the dynamic failure stress-strain curves. The characteristics of anisotropy are important factors that lead to the long plastic deformation stage and curve fluctuation of raw coal. These results showed that the dynamic mechanical properties of structural anisotropic coal rock are obviously different, which can be defined as anisotropic characteristics. However, only few lectures focus on the dynamics mechanical properties of structural anisotropic coal under low strain rate.

In order to reveal the dynamic mechanical properties of coal body in deep hole blasting under the different distances and directions, the coal samples in three coring directions (parallel to the bedding direction, oblique 45^o^ to bedding direction and perpendicular to the bedding direction) were tested by the self-made vertical SHPB impact device under low strain rate and five kinds of impact loads, which ensure the integrity of coal samples and provide the theoretical basis for the next permeability test.

## 2 The preparation of coal samples

**Fig 1:**
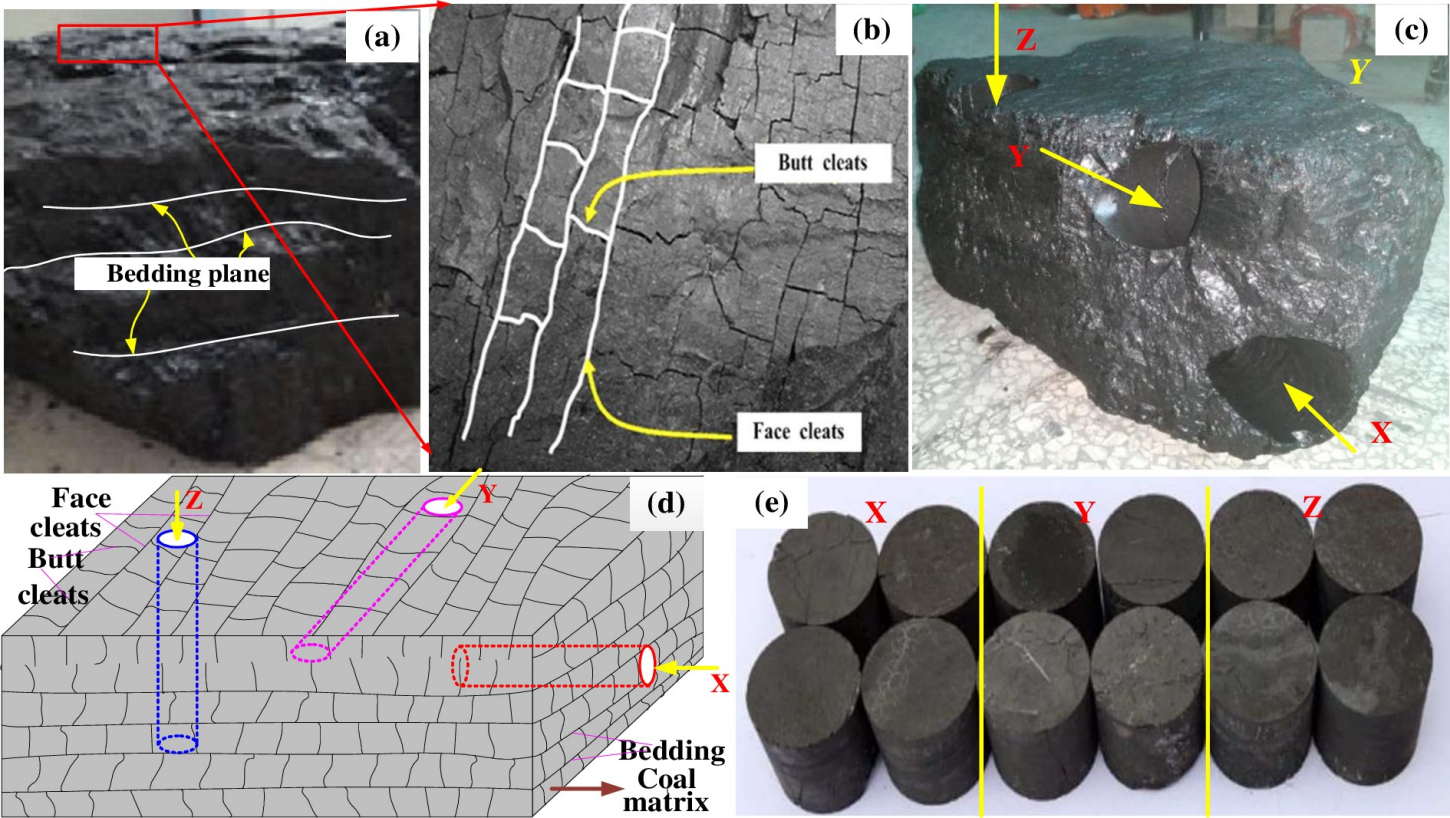
Preparation of coal samples (a) Schematic diagram of bedding plane (b) Enlarged diagram of butt cleats and face cleats (c) Coal block after three coring directions(d) Schematic diagram of three coring directions (e) Some coal samples.

The coal samples of Yangquan mine were studied in the laboratory, which provided the theoretical basis and service for the study of dynamic mechanical properties. No specific license is required for Yangquan mine, and no endangered or protected species are involved in the sampling process. The coal samples were taken from No.2 coal mine in Yangquan, Shanxi Province, China. They are all primary structural coal. The water content (1.27%), ash content (18.46%), and volatile matter (9.34%) of coal were determined. The coal metamorphic degree is anthracite. According to the ministry standard of coal industry of the people's Republic of China (MT49-1987), the drop hammer method is a common method to measure the consistent coefficient of coal, and the average consistent coefficient of coal samples is 1.21, as shown in Table 1. The bedding and cleat of coal body are obvious, which could be easily distinguished in the sampling process, as shown in Fig 1A and 1B. In this study, the coal samples were cored in parallel to the bedding direction (*θ*=0°, represented by symbol X), oblique 45^o^ to bedding direction (*θ*=45°, represented by symbol Y), and perpendicular to the bedding direction (*θ*=90°, represented by symbol Z), as shown in Fig 1C and 1D.

The diameter and height of coal samples are 50 mm. The 45 coal samples were tested for dynamic impact test (5 impact loads), and 3 coal samples were taken for each impact load. Some coal samples are shown in Fig 1E.

**Table 1:** The measured value of consistent coefficient.

Measured value			The average value
1	2	3	1.21
1.17	1.24	1.22	

## 3 The basic mechanical property parameters of coal samples

The uniaxial compression strength (UCS), Brazilian disc test (BDT) and variable angle shear tests were carried out to determine the mechanical parameters of coal samples, such as the elastic modulus *E*, Poisson's ratio *μ*, uniaxial compressive strength *σ_c_*, uniaxial tensile strength *σ_t_*, shear strength *τ_f_*, and internal friction angle *φ*. In the uniaxial compression test, the axial and transverse strain of coal samples were measured by the resistance strain gauge and YE2539 static strain gauge. Brazilian disc test and variable angle shear test were completed with corresponding clamps, respectively.

The mechanical property parameters of coal samples are shown in Table 2. According to the different coring directions, it can be seen that the average compressive strength in the Z direction is maximum and in the Y direction is minimum.

**Table 2:** The mechanical property parameters of coal samples.

Direction	*ρ*/(g/cm^3^)	*E*/GPa	*μ*	*σ*_*c*_/MPa	*σ*_*t*_/MPa	*τ*_*f*_/MPa	*φ*/
Z direction	1.417	3.972	0.295	25.37	1.273	7.41	20.35
Y direction	1.415	3.812	0.327	13.13	1.096	7.216	18.79
X direction	1.422	3.778	0.333	14.82	0.985	7.165	17.92

## 4 Dynamic impact test of coal samples

### 4.1 Experimental system

The self-made vertical SHPB impact device was adopted, with a similar principle to the horizontal impact SHPB device. The driving device is the only difference, and the driving device of horizontal SHPB impact device is pneumatic loading, while the driving device of vertical SHPB impact device is gravity loading. For horizontal SHPB impact device, the higher the gas pressure is, the more stable the impact speed is. On the contrary, the smaller the gas pressure is, the more unstable the impact speed is. Therefore, there is a minimum gas pressure value for the bullet to exit the chamber. The vertical SHPB impact device without high-pressure gas is easy to operate at low speed, and its safety is better than that of horizontal SHPB impact device. Comparing with the drop hammer device, the self-made vertical SHPB impact device has the following advantages in: 1) investigating the stress-strain relationship under low speed and low strain rate 2) avoiding the difficulty of directly measuring the stress or strain of the samples under the impact load. 3) calculating indirectly the dynamic mechanical constitutive relationship of the samples. However, the dynamic constitutive relation of the samples cannot be obtained by the drop hammer device.

**Fig 2:**
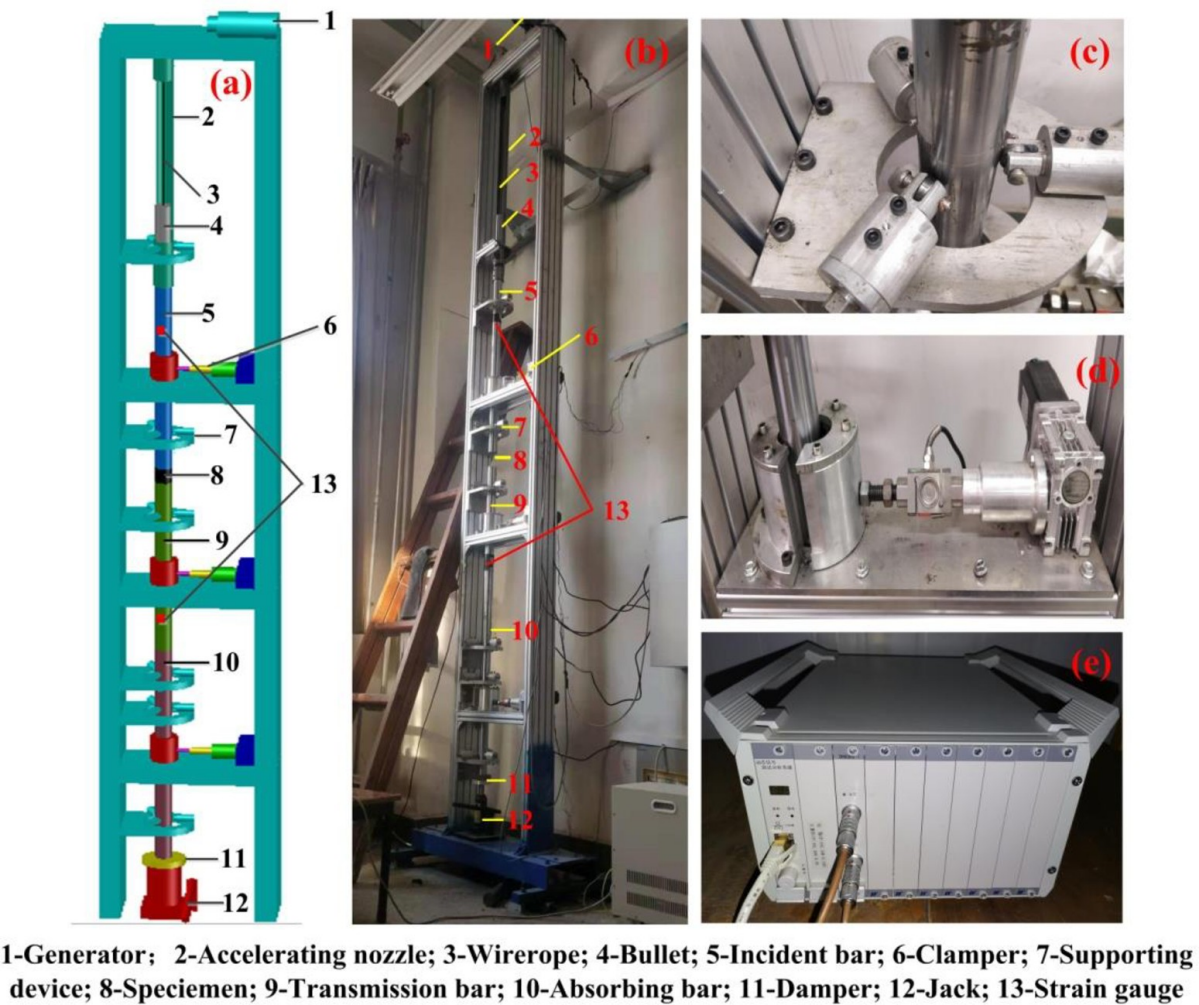
Self-made vertical SHPB impact device (a) Schematic diagram (b) Impact test system (c) Partial enlarged drawing of support device (d) Partial enlarged drawing of clamper (e) Dh8302-1 dynamic tester.

The self-made vertical SHPB device [30] is mainly composed of the bullet (30 mm in length), incident bar (1000 mm), transmission bar (1000 mm), absorbing bar (700 mm), damper, Jack, clamp holder (in Fig 2D), supporting device (in Fig 2C) and strain gauge, as shown in Fig 2. The incident bar, transmission bar, and absorbing bar are all elastic bars, and the diameter of the bullet and the three elastic bars are 50 mm.

There is a scale on the side wall of the accelerating conduit 2, which could adjust the impact load. First of all, the generator 1 lifts the bullet 4 through the wire rope 3. After that, when the bullet 4 is released freely at a certain height, it will impact the incident bar. Finally, the strain gauge 13 will immediately generate a pulse signal and transmit it to the dynamic strain gauge (Fig 2E). In order to avoid the inaccurate test results caused by the shaking of the elastic bars during the impact process, the confining pressure applied on the three elastic bars is adjusted to ensure that all bars are in a fixed state before and after the impact. The support device 7 is used to regulate whether the sample is in line with the central axis of the elastic bars during the test. In order to reduce the friction effect, the vaseline was applied to lubricate the interface between the elastic bars and the coal sample.

### 4.2 Experiment principle

The density of the elastic bars used in the test is 7850 kg/m^3^, the elastic modulus of the elastic bars is 210 GPa, and the speed of wave propagation in the elastic bars is 5.19 km/s.

The impact velocity *v* of the bullet is obtained as

$$v=\sqrt{2gH}$$ (1)

Where *g* is the acceleration of gravity, 9.8 m/s^2^; *H* is the free-falling height of the bullet's center of gravity, m.

The impulse of the bullet is obtained as

$$I=Ft=mv-mv_{0}$$ (2)

Where *F* is the resultant force of all external forces including gravity, *N*; *t* is the impact time, s; *m* is the mass of the bullet, *kg*; *v* and *v_0_* are the end velocity and initial velocity of the bullet, m/s.

According to Eq (1), the impulse value can be obtained under the *H*, as shown in Table 3. According to the impact load, the coal samples are divided into five groups, and coal samples in three coring directions are taken as a group. Dynamic impact test was carried out from small impulse to large impulse.

**Table 3:** The parameters of impact test.

Number	Impact height/m	Coal sample number	Impact velocity/m·s^-1^	Impulse/kg·m·s^-1^
1	0.25	Z1-α、Z1-β、Z1-γ	2.21	10.21
		Y1-α、Y1-β、Y1-γ		
		X1-α、X1-β、X1-γ		
2	0.4	Z2-α、Z2-β、Z2-γ	2.8	12.94
		Y2-α、Y2-β、Y2-γ		
		X2-α、X2-β、X2-γ		
3	0.55	Z3-α、Z3-β、Z3-γ	3.28	15.16
		Y3-α、Y3-β、Y3-γ		
		X3-α、X3-β、X3-γ		
4	0.7	Z4-α、Z4-β、Z4-γ	3.7	17.1
		Y4-α、Y4-β、Y4-γ		
		X4-α、X4-β、X4-γ		
5	0.85	Z5-α、Z5-β、Z5-γ	4.08	18.86
		Y5-α、Y5-β、Y5-γ		
		X5-α、X5-β、X5-γ		

According to the simplified “three-wave equation”, the stress, strain, and strain rate can be obtained as follows:

$$\sigma (t)=E\frac{A}{A_{0}}\varepsilon_{t}(t)$$ (3)

$$\dot{\varepsilon }(t)=-\frac{2C}{L_{0}}\varepsilon_{r}(t)$$ (4)

$$\varepsilon (t)=-\frac{2C}{L_{0}}\int_{0}^{t}\varepsilon_{r}(t)dt$$ (5)

In the Eq (3)~Eq (5), $\dot{\varepsilon }(t)$, *ε*(*t*) and *σ*(*t*) are loading strain rate, strain and loading stress of coal samples respectively; *ε*_*r*_(*t*) and *ε*_*t*_(*t*) are reflected wave strain and transmitted wave strain in the compression bar respectively; *E*, *C* and *A* are elastic modulus, wave velocity and cross-sectional area of the compression bar respectively; *A*_0_ and *L*_0_ are cross-sectional area and the length of the sample respectively.

## 5 Impact test results and discussions

### 5.1 Test signal characteristics and denoising

**Fig 3:**
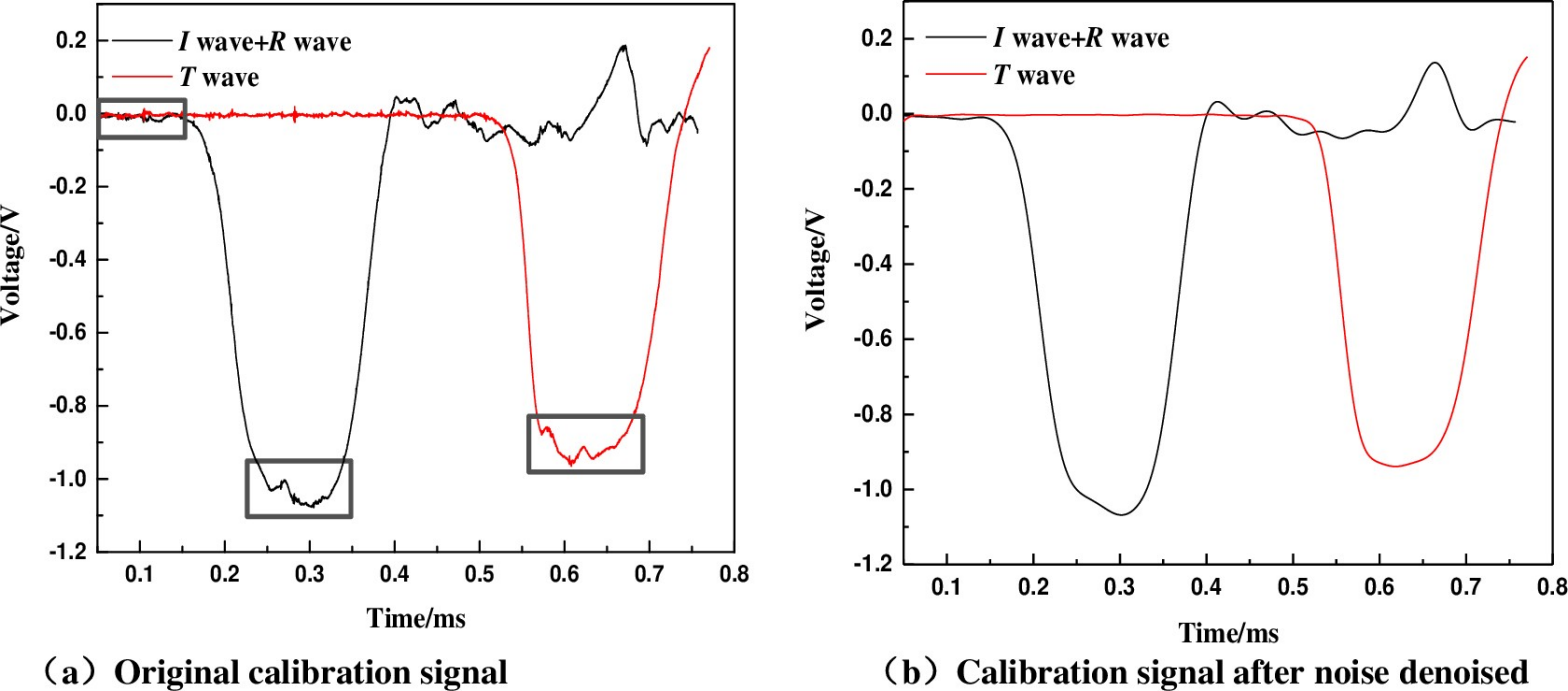
Calibration waveform curves of empty bar.

In the experiment, the shape of the input wave is changed by using the copper strip as shaping, and the friction effect of the end face is weakened by using the lubricant. Herein, the Fourier transform was used to denoise the test signal. The filtering effect in Fig 3 is taken as an example to illustrate the denoising result of the calibration signal. The original signals and denoising signals of the incident wave(*I* wave), reflected wave (*R* wave) and transmitted wave (*T* wave) measured in calibration test (falling height is 4m) are shown in Fig 3A and 3B. It can be seen from Fig 3A that the original signal is affected by noise, which needs to be denoised. The incident bar generates a pulse reflection signal with a low peak value, which approximately meets the one-dimensional stress assumption. As shown in Fig 3B that the peak value of the filtered signal is almost unchanged, and the noise has been filtered out. The denoised waveform is beneficial for data processing and analysis.

Under the impact test, the deformation and failure process of coal body is generally manifested as pore shrinkage, coal body compaction, particle contact area enlargement, fracture group formation, and partial interregional adhesion reduction, etc.

### 5.2 The strain rate

**Fig 4:**
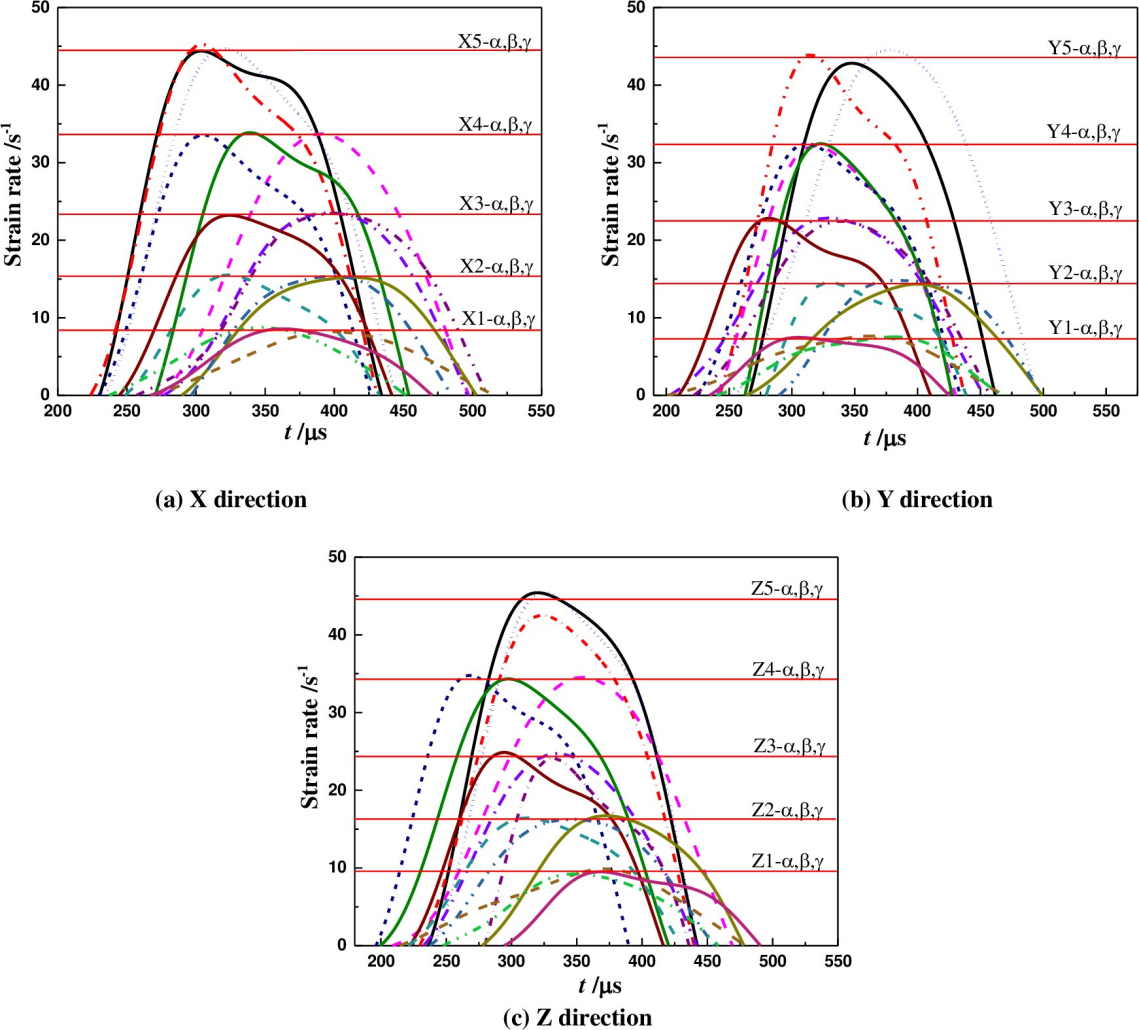
Strain rate variation of coal samples in different coring directions.

**Fig 5:**
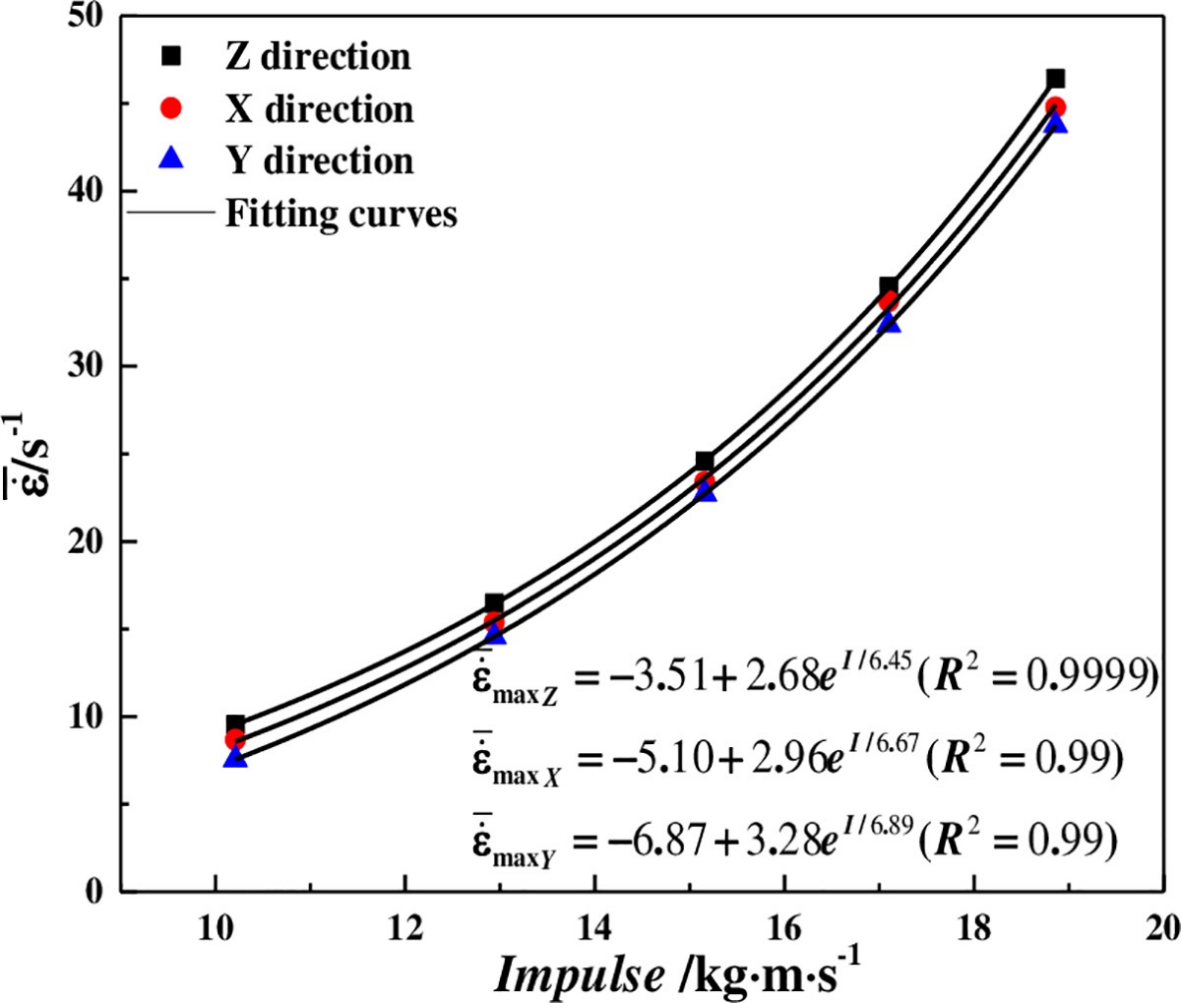
The relationship between impulse and average maximum strain rate.

According to Eq (4), the strain rate curves under impact load can be calculated, as shown in Table 4, Figs 4 and 5. The maximum strain rate (${\dot{\varepsilon }}_{\max}$) increases exponentially with impact load in the same coring directions, and the correlation coefficients are above 0.99. Moreover, under the same impact load, the maximum strain rate is the largest in the Z direction, middle in the X direction, and smallest in the Y direction. However, the overall differences are not significant.

**Table 4:** The average maximum strain rates of coal samples.

*I /*kg·m·s^-1^	*θ*=90°	${\dot{\varepsilon }}_{\max}$/s^-1^	${\bar{\dot{\varepsilon }}}_{\max}$/s^-1^	*θ*=0°	${\dot{\varepsilon }}_{\max}$/s^-1^	${\bar{\dot{\varepsilon }}}_{\max}$/s^-1^	*θ*=45°	${\dot{\varepsilon }}_{\max}$/s^-1^	${\bar{\dot{\varepsilon }}}_{\max}$/s^-1^
10.21	Z1-*α*	10.087	9.545	X1-*α*	8.017	8.682	Y1-*α*	7.561	7.561
	Z1-β	9.534		X1-β	8.454		Y1-β	8.044	
	Z1-γ	9.015		X1-γ	9.576		Y1-γ	7.078	
12.94	Z2-*α*	16.213	16.474	X2-*α*	15.112	15.375	Y2-*α*	14.339	14.569
	Z2-β	17.054		X2-β	15.065		Y2-β	14.812	
	Z2-γ	16.156		X2-γ	15.948		Y2-γ	14.556	
15.16	Z3-*α*	25.316	24.568	X3-*α*	24.015	23.389	Y3-*α*	22.271	22.712
	Z3-β	24.323		X3-β	23.238		Y3-β	22.047	
	Z3-γ	24.066		X3-γ	22.914		Y3-γ	23.817	
17.1	Z4-*α*	34.043	34.551	X4-*α*	34.021	33.714	Y4-*α*	31.815	32.361
	Z4-β	34.027		X4-β	33.755		Y4-β	32.726	
	Z4-γ	35.583		X4-γ	33.366		Y4-γ	33.542	
18.86	Z5-*α*	47.335	46.421	X5-*α*	44.377	44.775	Y5-*α*	42.815	43.754
	Z5-β	46.512		X5-β	45.231		Y5-β	43.942	
	Z5-γ	45.416		X5-γ	44.717		Y5-γ	44.505	

### 5.3 The characteristics of the stress-strain curve

**Fig 6:**
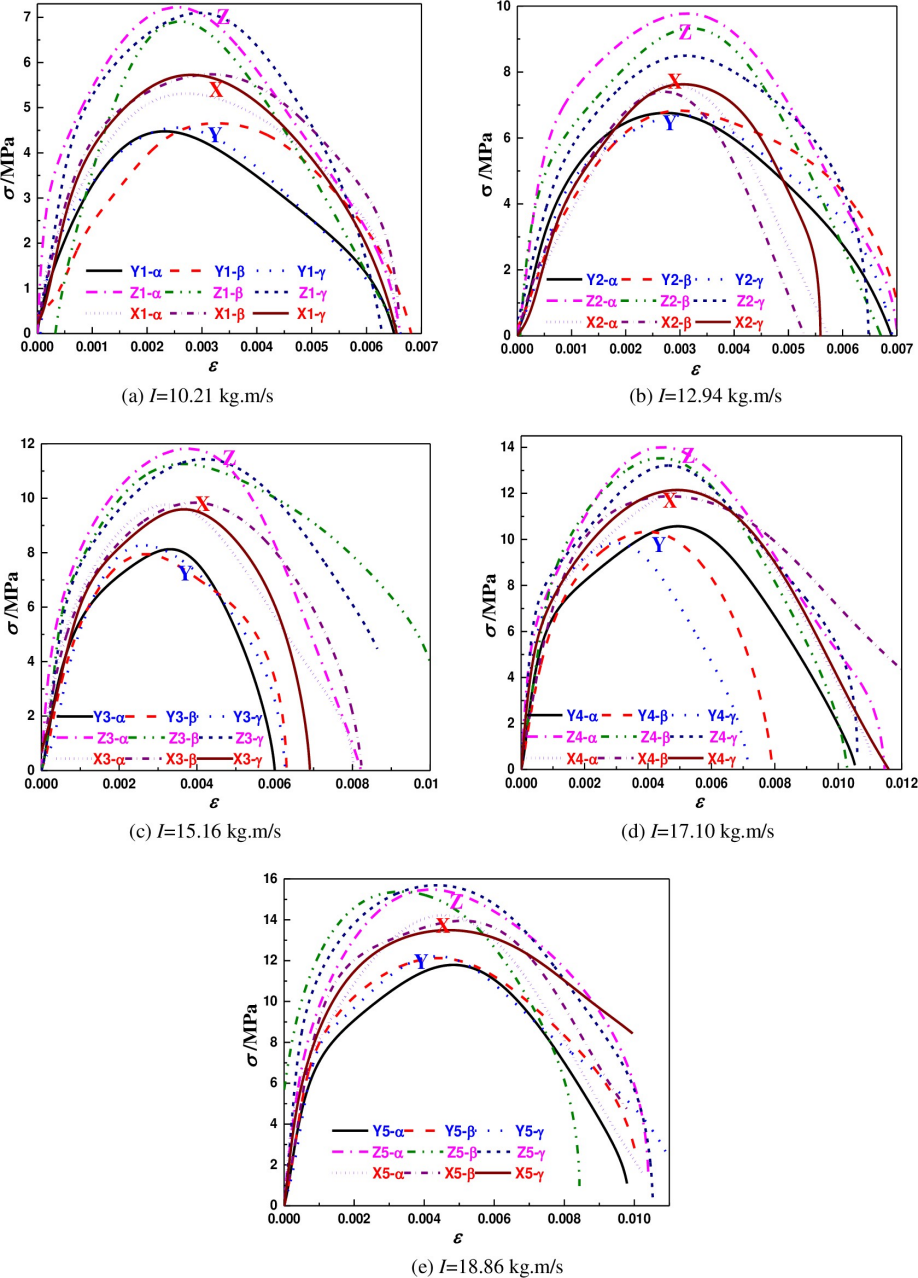
Stress-strain curves of coal samples under different impulses.

Under different impulses, the stress-strain curves of coal samples can be calculated according to Eq (3), Eq (5), as shown in Fig 6 and Table 4. The stress-strain curve can be roughly divided into linear elastic stage, plastic deformation stage and failure stage. The curve almost has no concave section and obvious initial compaction stage, but directly enters the linear elastic stage. The stress increases linearly with the strain approximately in the initial stage of strain. The reason is that the coal samples are relatively dense, the internal micro-cracks are not well developed. Under the impact load, the macroscopic and microscopic defects of the coal samples are too late to close, and the compaction stage is very short.

As shown in Fig 6, the stress rises rapidly with the strain rate. The curve basically has no plastic plateau, which indicates that the coal samples show obvious brittleness with the change of strain rate. When the stress value rises to 80% of the ultimate strength, the upward trend of the curve slows down, showing a similar strengthening stage to that of rock [31,32]. The degree of slowing down is related to the number of cracks, indicating that the primary cracks start to close, and the strain growth rate is accelerated. This phenomenon will appear in coal rock with internal cracks [33].

Fig 6 shows that the peak stress is obviously different in three coring directions. Under the same impulse, the peak stress of coal samples in the Z direction is the maximum, and the ${\bar{\sigma }}_{\max}$ value is 15.506 MPa. The peak stress is the minimum in the Y direction, and the ${\bar{\sigma }}_{\max}$ value is 12.072 MPa. The average values of the peak stress are shown in Table 5.

**Table 5:** The average peak stress of coal samples.

*I*/kg·m·s^-1^	*θ*=90°	*σ*_max_/MPa	${\bar{\sigma }}_{\max}$/MPa	*θ*=0°	*σ*_max_/MPa	${\bar{\sigma }}_{\max}$/MPa	*θ*=45°	*σ*_max_ /MPa	${\bar{\sigma }}_{\max}$/MPa
10.21	Z1-α	7.225	7.077	X1-α	5.306	5.574	Y1-α	4.472	4.555
	Z1-β	6.909		X1-β	5.701		Y1-β	4.638	
	Z1-γ	7.098		X1-γ	5.715		Y1-γ	4.556	
12.94	Z2-α	9.774	9.198	X2-α	7.540	7.522	Y2-α	6.758	6.749
	Z2-β	9.325		X2-β	7.401		Y2-β	6.756	
	Z2-γ	8.496		X2-γ	7.626		Y2-γ	6.732	
15.16	Z3-α	11.822	11.479	X3-α	9.763	9.73	Y3-α	8.116	8.105
	Z3-β	11.184		X3-β	9.837		Y3-β	7.936	
	Z3-γ	11.432		X3-γ	9.591		Y3-γ	8.263	
17.1	Z4-α	14.006	13.582	X4-α	12.115	12.041	Y4-α	10.573	10.241
	Z4-β	13.528		X4-β	11.861		Y4-β	10.318	
	Z4-γ	13.213		X4-γ	12.148		Y4-γ	9.832	
18.86	Z5-α	15.481	15.506	X5-α	14.220	13.882	Y5-α	11.788	12.072
	Z5-β	15.356		X5-β	13.952		Y5-β	12.213	
	Z5-γ	15.681		X5-γ	13.473		Y5-γ	12.214	

**Fig 7:**
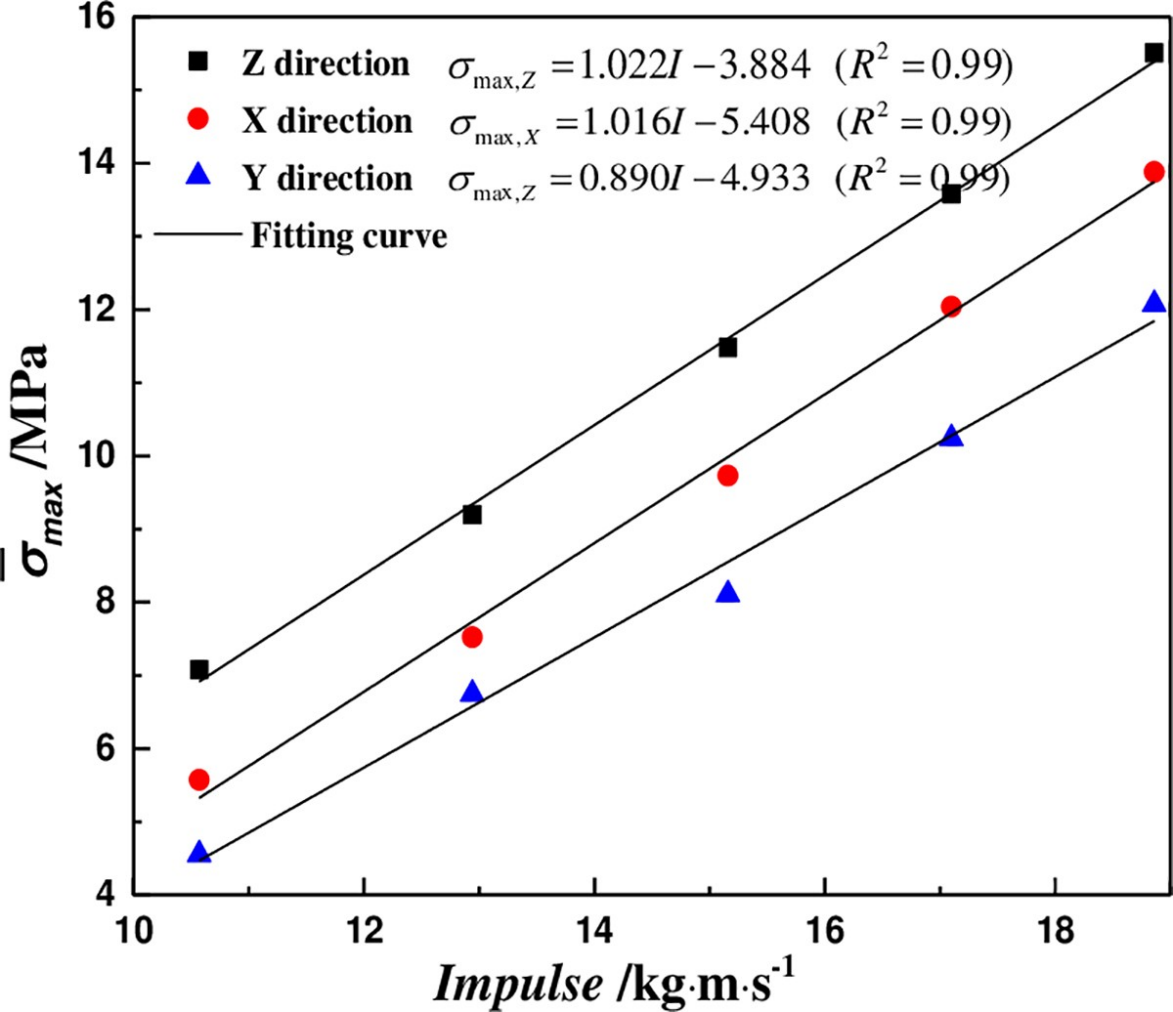
The relationship between the impulse and average peak stress.

Fig 7 shows the relationship between the impulse and average peak stress in three coring directions. The peak stress increases linearly with the impact load, and the correlation coefficients are all above 0.98. Obviously, the impact load and structural anisotropy have significant influence on the peak stress of coal body.

### 5.4 Dynamic Intensity Growth Factor (DIF)

In order to compare the growth of dynamic strength of coal body with that of static strength under different impact loads, the ratio of dynamic strength to static strength is defined as Dynamic Intensity Growth Factor (DIF), that is:

$$DIF=\frac{\sigma_{d}}{\sigma_{c}}$$ (6)

**Fig 8:**
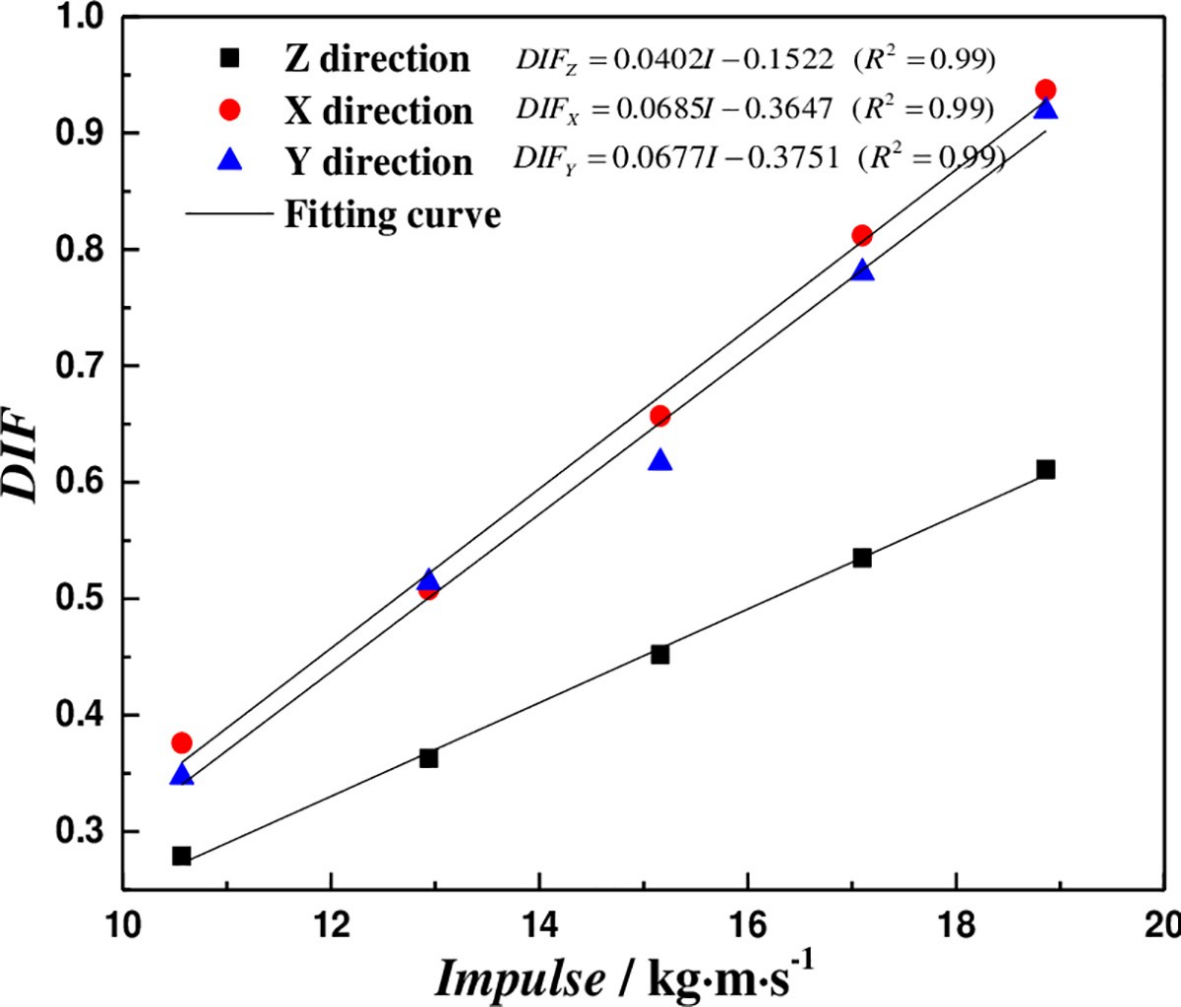
Variation of the DIF with the impulse.

Where, *σ*_*d*_ and *σ*_*c*_ are dynamic and static compressive strength of coal samples respectively. The DIF can be calculated *via* Eq (6) and data of Table 5, as shown in Table 6. The relationship between DIF and impulse is fitted, as shown in Fig 8.

**Table 6:** The average value of DIF under different impulses.

Impulse /kg·m·s^-1^	*θ*=90°	DIF	*θ*=0°	DIF	*θ*=45°	DIF
10.21	Z1-α	0.279	X1-α	0.376	Y1-α	0.347
	Z1-β		X1-β		Y1-β	
	Z1-γ		X1-γ		Y1-γ	
12.94	Z2-α	0.363	X2-α	0.508	Y2-α	0.514
	Z2-β		X2-β		Y2-β	
	Z2-γ		X2-γ		Y2-γ	
15.16	Z3-α	0.452	X3-α	0.657	Y3-α	0.617
	Z3-β		X3-β		Y3-β	
	Z3-γ		X3-γ		Y3-γ	
17.1	Z4-α	0.535	X4-α	0.812	Y4-α	0.780
	Z4-β		X4-β		Y4-β	
	Z4-γ		X4-γ		Y4-γ	
18.86	Z5-α	0.611	X5-α	0.937	Y5-α	0.919
	Z5-β		X5-β		Y5-β	
	Z5-γ		X5-γ		Y5-γ	

Fig 8 shows that the DIF of coal samples increases linearly with the impact load in three coring directions. The dynamic compressive strength does not exceed the static compressive strength, which due to the fact that the coal samples are not broken, and only some microcracks and internal damage are produced in the coal body [34].

### 5.5 Dynamic elastic modulus

The dynamic stress-strain curve of coal rock is the arc, and secant modulus is used instead of elastic modulus to reflect the average stiffness of coal rock.

$$E_{50}=\frac{\sigma_{d50}}{\varepsilon_{d50}}$$ (7)

**Fig 9:**
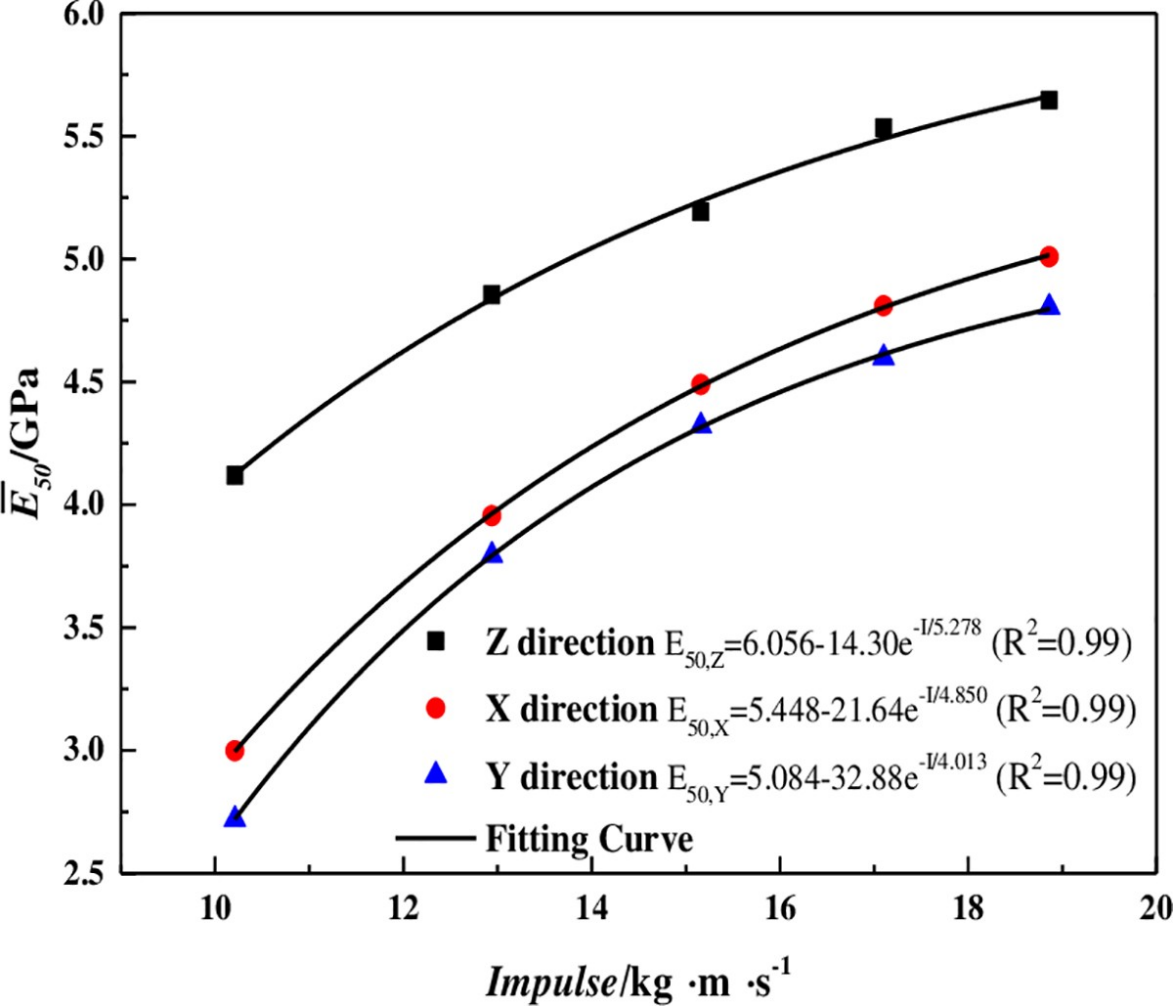
The relationship between impulse and dynamic elastic modulus.

Where, *σ*_*d*50_ is the stress value when the dynamic compressive strength peaks at 50%, *ε*_*d*50_ is the corresponding axial strain. The dynamic elastic modulus is obtained by processing the measured stress-strain curve, as shown in Table 7. The relationship between the average dynamic elastic modulus and impulse in three coring directns is shown in Fig 9.

**Table 7:** The dynamic elastic modulus under different impulses.

*I* /kg·m·s^-1^	*θ*=90°	*E*_50_/GPa	${\bar{E}}_{50}$/GPa	*θ*=0°	*E*_50_/GPa	${\bar{E}}_{50}$/GPa	*θ*=45°	*E*_50_/GPa	${\bar{E}}_{50}$/GPa
10.21	Z1-α	4.761	4.119	X1-α	3.008	2.998	Y1-α	3.096	2.719
	Z1-β	3.647		X1-β	2.817		Y1-β	2.104	
	Z1-γ	3.950		X1-γ	3.169		Y1-γ	2.958	
12.94	Z2-α	5.465	4.855	X2-α	3.969	3.955	Y2-α	4.172	3.794
	Z2-β	4.612		X2-β	4.001		Y2-β	3.561	
	Z2-γ	4.487		X2-γ	3.894		Y2-γ	3.649	
15.16	Z3-α	5.471	5.192	X3-α	4.926	4.489	Y3-α	4.057	4.321
	Z3-β	5.403		X3-β	4.247		Y3-β	4.357	
	Z3-γ	4.701		X3-γ	4.295		Y3-γ	4.549	
17.1	Z4-α	5.445	5.534	X4-α	4.969	4.809	Y4-α	3.548	4.598
	Z4-β	5.344		X4-β	4.995		Y4-β	4.441	
	Z4-γ	5.825		X4-γ	4.463		Y4-γ	5.804	
18.86	Z5-α	6.426	5.646	X5-α	4.909	5.009	Y5-α	3.967	4.804
	Z5-β	5.013		X5-β	4.958		Y5-β	4.773	
	Z5-γ	5.499		X5-γ	5.159		Y5-γ	5.521	

As can be seen from Fig 9, the dynamic elastic modulus increases exponentially with the impact load, and the correlation coefficients are all greater than 0.98.

## 6 Failure characteristics of coal samples

**Fig 10:**
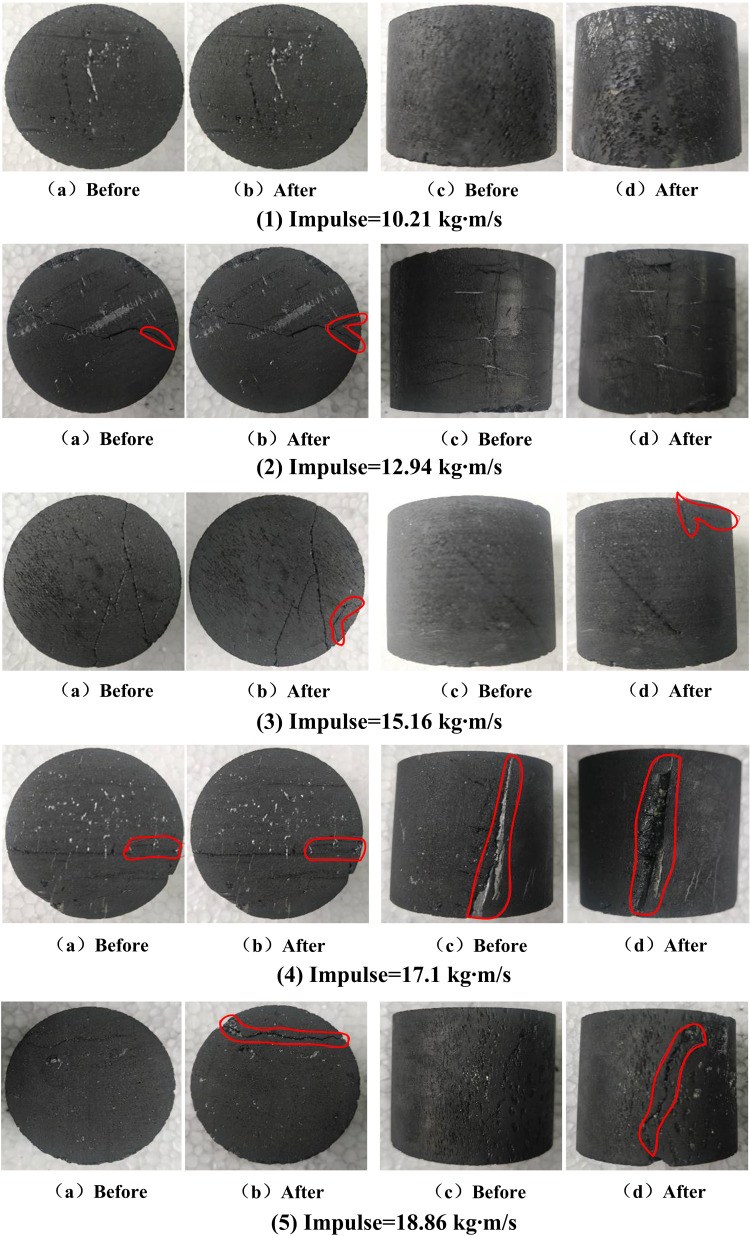
Failure mode of coal samples under different impulses.

Fig 10 shows the different failure mode characteristics of coal samples under different impulses. The failure degree of coal samples increases with the impulse, the transformation from axial splitting failure state to radial crushing failure state shows the strain rate effect. There are many cracks in the coal samples. The nature of cracks indirectly determines the failure modes of the coal samples, which are caused by the generation and expansion of the internal cracks [34,35]. Under the lower impact load, the stress wave passes through the crack, which makes the original crack extend and expand. More energy is needs to generate a new crack. When the impact load is small, the energy generated is not enough to produce a new crack, and the development of the new crack is not obvious. Therefore, the stress concentration will appear at the crack tip to make it crack first under stress wave, then the crack will expand along the tip, and finally the coal samples will be split axially. When the coal samples are subjected to high impact load, due to the existence of shear pressure, the coal samples will show brittleness. Some new cracks will be generated under the effect of stress wave, which will extend and penetrate, and the internal cracks in the coal samples will continue to increase. Therefor the surface of the coal samples will show cracks, a small number of broken and other phenomena.

## 7 Conclusion

The dynamic stress-strain, dynamic elastic modulus and dynamic intensity growth factor properties of raw coal samples with three coring directions were studied by using the self-made vertical SHPB device under low strain rate and five kinds of impact loads. The conclusions are as follows:

1. The self-made vertical SHPB device is suitable for the loading of coal samples at low speed and low strain rate.
2. Under same coring direction, the strain rate increases exponentially with the impact load. The strain rate is weakly affected by the structural anisotropic coal.
3. The dynamic stress-strain characteristics of coal samples show great differences in directions. Under the same impact load, the average peak stress of coal samples is maximum in the perpendicular to the bedding direction, minimum in the oblique 45^o^ to bedding direction, displaying the anisotropic characteristics of coal structure.
4. The dynamic elastic modulus and the dynamic intensity growth factor of the coal samples increase with the impact load, and the correlation coefficients are greater than 0.98, showing the correlation and sensitivity to the impact load.
